# Small-Cell Type, Poorly Differentiated Neuroendocrine Carcinoma of the Gallbladder: A Case Report and Review of the Literature

**DOI:** 10.1155/2019/8968034

**Published:** 2019-04-01

**Authors:** Kishore Kumar, Hassan Tariq, Rafeeq Ahmed, Chime Chukwunonso, Masooma Niazi, Ariyo Ihimoyan

**Affiliations:** ^1^Department of Medicine, Bronxcare Health System, Bronx, New York, USA; ^2^Division of Gastroenterology, Bronxcare Health System, Bronx, New York, USA; ^3^Department of Pathology, Bronxcare Health System, Bronx, New York, USA

## Abstract

The poorly differentiated small-cell type, neuroendocrine carcinoma (NEC) of the gallbladder is a very uncommon subtype of a neuroendocrine tumor of the gastrointestinal tract. Nonsecretory NEC by virtue of its nonspecific and subtle clinical presentation of the tumor is usually diagnosed at an advance stage with presenting symptoms related to either locally advance disease or from metastatic disease. Though the radiologic imaging does identify the gall bladder cancer, the tumor lacks a specific diagnostic test; therefore, the diagnosis is almost always confirmed on histopathologic and immunohistochemical staining. We present a case of a poorly differentiated, small-cell neuroendocrine tumor of the gallbladder. The patient died within 3 months after the definitive diagnosis was made. Survival from this deadly malignancy can be improved with aggressive surgical treatment followed by chemotherapy and radiotherapy on a case-by-case scenario. The systemic chemotherapy remained the treatment of choice for an unresectable tumor (Chen et al., 2014).

## 1. Introduction

Primary neuroendocrine tumors (NETs) are distinct tumors with characteristic histological features. They commonly involve the gastrointestinal tract (73.7%) or bronchopulmonary system (25.1%). They arise from enterochromaffin cells and their precursor neuroendocrine cell type. NETs can be found in any location of the body; however, as mentioned above, they are commonly seen to involve gastrointestinal and pulmonary systems. There are no neuroectodermal cells in the gallbladder; hence, the primary neuroendocrine tumor of the gallbladder hypothetically arises from a pleuropotent stem cell or gallbladder lining metaplasia due to chronic insult caused by the gallstone.

In general, the type of the neuroendocrine tumor (NET) is determined by tumor differentiation (the extent to which neoplastic cells resemble their nonneoplastic normal cell) and histologic grade of the tumor (the proliferative activity of the tumor commonly measured by the mitotic rate and Ki-67 index). The World Health Organization (WHO) classified NETs into two broad subgroups [1]. Well-differentiated NETs further subdivide into low grade (G1) and intermediate grade (G2) based on the proliferative rate (mitotic rate and Ki-67 index). Clinically, low and intermediate grade NETs have indolent behavior [2]. Poorly differentiated neuroendocrine carcinoma (G3) is high grade in nature due to an accelerated mitotic rate and Ki-67 index (Table 1). Clinically, poorly differentiated neuroendocrine carcinoma of the gallbladder is an aggressive tumor. According to the AJCC (American Joint Committee on Cancer), NETs are staged as a tumor (T), node (N), and metastasis (M) staging system.

A population-based study by Kanakala et al. has suggested that the small intestine is the most common site (44.7%), followed by the rectum (19.6%). Primary neuroendocrine tumors of the gallbladder are rare (0.2-1%) [1]. Peptide nonsecretory gallbladder NETs are more common than secretory NETs and may present with symptoms of either local disease or metastasis [3].

As with other tumors of the gall bladder, the small-cell type NEC is more prevalent in women, associated with a gallstone, and mean age at the time of diagnosis is ~64 years. Overall, the small-cell neuroendocrine carcinoma of the gallbladder is extremely rare and carries a poor prognosis with survival worse than adenocarcinoma of the gallbladder due to advanced disease at the time of diagnosis and highly malignant nature of the tumor.

## 2. Case Presentation

A 61-year African woman with no comorbidities presented to the emergency department with worsening of right upper quadrant abdominal pain for 3 weeks. She described the pain as constant, nonradiating, 10/10 in intensity, relieved with paracetamol, and associated with subjective weight loss and decreased appetite. The patient arrived 2 weeks ago to visit her children in the USA. According to the patient, she had a similar episode of pain approximately 6 months ago in Nigeria, which was treated symptomatically by a local physician, and pain subsided in the next few days with analgesics. She reports no associated symptoms of nausea, vomiting, constipation, diarrhea, flushing, wheezing, cough, melena, hematochezia, or a headache. She never had any surgery, and she does not smoke, drink alcohol, or use drugs. She has no family history of cancer. Physical examination revealed normal vital signs except her blood pressure was elevated to 183/103 mmHg. Her abdominal examination showed mild hepatomegaly and tenderness at the right upper quadrant with no guarding or rigidity. The rest of the physical examination was unremarkable. She underwent routine blood tests and ultrasound of the abdomen in the emergency department, which showed a heterogenous liver mass in the right hepatic lobe; other masses in the region of the pancreas and gallbladder were not clearly identified; hence, the patient was admitted for further evaluation. Her laboratory investigation revealed mild microcytic anemia with hemoglobin of 11.7 mg/dl, elevated alpha-fetoprotein of 48.77 ng/ml (normal value less than 6.1 ng/ml), and an increased level of the carcinoembryonic antigen of 36.4 (normal value less than 5 ng/ml). Otherwise, she had normal liver function tests and serum creatinine along with the normal level of cancer antigen 125 and cancer antigen 19-9. Her human immune deficiency virus (HIV), hepatitis B, and hepatitis C serologies were negative.

**Figure 1:**
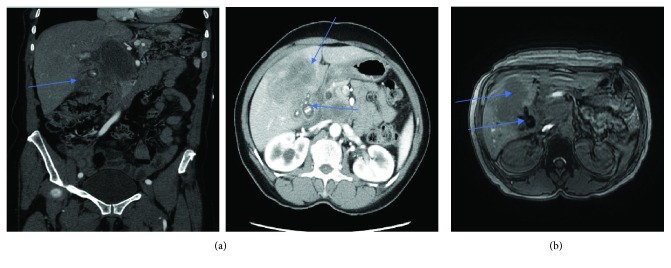
(a) CT of the abdomen coronal and axial views. A complex mass with foci of calcification in gallbladder fossa and 9 cm heterogeneous mass in the right hepatic lobe. (b) MRI of the abdomen axial view: large gallbladder complex mass of 5 × 4 cm with engulfed calcifications and stones extending into the liver. 9 cm liver mass with some central necrosis and calcifications and loss of clear fat plane separation between the gallbladder and right liver, associated with large hilar lymphadenopathy (4 × 5 cm) and compressive effects on the distal biliary system.

**Figure 2:**
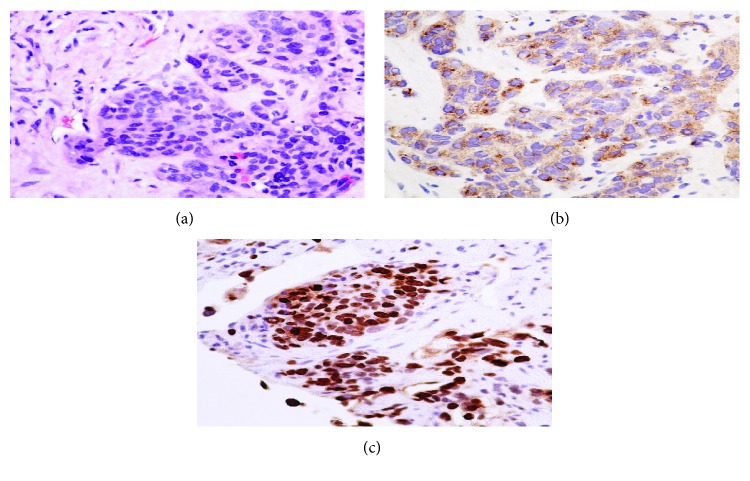
(a) Neuroendocrine carcinoma, small-cell type, comprised of tumor cells arranged in an organoid pattern with peripheral palisading. There is cell compression with molding and increased mitosis. H&E: magnification ×400. (b) Neuroendocrine carcinoma, small-cell type; the tumor cells are immunoreactive to immunomarker synaptophysin. Immunohistochemical stain: ×400. (c) Neuroendocrine carcinoma, small-cell type. Proliferating marker Ki-67 shows more than 90% intranuclear positivity. Immunohistochemical stain: ×400.

Computed tomography (CT) of the abdomen and pelvis with contrast (Figure 1(a)) showed a solid heterogeneous mass of 9 cm in the right hepatic lobe, and a 5 cm complex mass with foci of calcification was identified in the gallbladder fossa associated with portacaval and porta hepatis lymphadenopathy measuring 3.5 cm in anteroposterior and extending 7 cm in the craniocaudal dimension. Magnetic resonance (MR) imaging of the abdomen (Figure 1(b)) delineates the same findings with a high likelihood of a gallbladder tumor with liver metastases. There was no evidence of mass or metastatic disease in the chest, bone, and brain. Subsequently, liver mass histopathology showed the poorly differentiated metastatic carcinoma favoring a small-cell type. Thereafter, on immunohistochemical staining, tumor cells were positive for cytokeratin AE1/AE3 and synaptophysin. The stain was equivocal for chromogranin-A. Tumor cells were negative for alpha-fetoprotein (AFP), cytokeratin 7 (CK07), cytokeratin 20 (CK20), cluster of differentiation 2 (CD2), cancer antigen 19-9 aka carbohydrate antigen 19-9 (CA19-9), and cancer antigen-125 (CA-125) antibodies (Figures 2(a)–2(c)). Based on the positive immunohistochemical staining for synaptophysin, the final diagnosis of poorly differentiated, small-cell type, neuroendocrine of carcinoma of gallbladder origin with liver metastasis was made. The tumor was deemed unresectable due to the locally advance invasion of the surrounding structure; hence, palliative chemotherapy was offered. The patient decided to travel back to her country and wished to pursue further care in her home country, Nigeria. On telephonic follow-up at 3 months, the daughter states that the patient passed away a few days back.

## 3. Discussion

Primary NETs can occur anywhere in the body wherever there are enterochromaffin cells, but the gallbladder is a rare site of occurrence (<1%) [1]. As reported by Chen et al., the NET of the gall bladder comprises 0.5% of overall NET incidence and constitutes 2% of the gall bladder cancers [4]. The term neuroendocrine carcinoma (NEC) distincts from the neuroendocrine tumor (NEC vs. NET) with respect to its poor differentiation and high tumor grade. The tumor grade as mentioned above correlates with the mitotic count and Ki-67 proliferation index. Gallbladder NETs can be classified into four broad histological categories based on tumor differentiation and grade: (1) well-differentiated NETs (typical carcinoid), (2) well-differentiated neuroendocrine carcinoma (atypical or malignant carcinoid), (3) poorly differentiated neuroendocrine carcinoma (high-grade carcinoma—small-cell/large-cell types), and (4) mixed exocrine-endocrine carcinomas. The term heterogeneous carcinoma has been used when more than one histological feature is present at the same time, for example, adenocarcinoma with neuroendocrine carcinoma.

The most common early manifestation is a vague upper abdominal pain. Functionally, carcinomas can be divided into secretory or nonsecretory based on the production of the peptide substance. The nonsecretory neuroendocrine carcinoma manifests as symptoms of local disease (such as abdominal pain, weight loss, and jaundice) or symptoms due to metastatic disease. The functionally active (secretory) neuroendocrine tumor can give rise to symptoms related to secretion of the different peptide in addition to symptoms of local or metastatic disease. The tumor can produce various types of the peptide such as serotonin, histamine, prostaglandins, vasoactive intestinal peptide (VIP), substance P, and glucagon, thus leading to diarrhea, flushing, and hyperglycemia [2].

It is almost impossible to ascertain the diagnosis of neuroendocrine carcinoma preoperatively solely based on radiologic imaging such as ultrasonography, computed tomography (CT) scan, magnetic resonance imaging (MRI), and positron emission tomography CT (PET-CT). Histopathologic examination with immunohistochemical staining is required to make a definitive diagnosis. The tumor can stain positive for synaptophysin (SYN) in 75% of neuroendocrine carcinomas followed by chromogranin (CGA). In cases of functionally active or secretory neuroendocrine carcinoma, urine 5-hydroxyindoleacetic acid (5-HIAA) and nuclear imaging studies (octreotide scintigraphy or MIBG) may be useful to diagnose and respond to therapy. Microscopically, poorly differentiated neuroendocrine carcinomas appeared as atypical, small to intermediate-sized cells growing in the form of large ill-defined aggregates, often with necrosis and prominent angioinvasion and/or perineural invasion with high mitotic (>20/10 HPF) and proliferation (Ki − 67 > 20%) index.

A tumor restricted to mucosa or submucosa (TIS or T1) requires cholecystectomy alone; however, locally advanced disease with no evidence of distant metastasis required lymphadenectomy possible hepatic resection in order to achieve a tumor-free margin in addition to cholecystectomy. The improved survival has been seen when surgical therapy followed by adjuvant chemotherapy has been used in patients with locally invasive NEC. Systemic chemotherapy remained the treatment of choice where the tumor is inoperable or metastasized and can be considered in cases where specimen margins contain tumor involvement [4]. The large-cell NEC of the gall bladder has a worse prognosis due to its decreased responsiveness to chemotherapy. Similarly, to lung SCLC, cisplatin or carboplatin and etoposide have been used as the primary chemotherapy treatment. The chemotherapeutic agents such as topotecan, irinotecan, taxanes, and gemcitabine have been reserved for salvage with variable success. Local radiation therapy is reserved for palliation of pain from metastatic bone disease and cord compression. Metastatic disease to the liver may be treated with radiofrequency ablation, transarterial chemoembolization (TACE), and transarterial-radioablation (TARE) for palliative intend. One of the reports from SEERs reported median survival of 9.8 months in 278 gall bladder NETs with 5-year survival of 0% [5]. The somatostatin analog has been used with variable outcomes. Overall, poorly differentiated-type NEC, elevated Ki-67 index, high mitotic rate, and tumor invasion to the local structure are the poor prognostic factors.

## 4. Conclusion

The asymptomatic nature of the early disease and advanced stage at the time of diagnosis is the composite outcome of aggressiveness of the tumor. The patients are often diagnosed at an advanced stage with lymph node or liver invasion hence carrying a poor prognosis. Multimodel therapy with surgical resection, chemotherapy, and radiotherapy has shown to increase survival in some studies; however, due to the low incidence of the disease and no universally accepted treatment protocol, further studies are needed to emphasize the treatment [6]. Review of current literature revealed a dismal 5-year survival rate for small-cell cancer.

## Figures and Tables

**Table 1:** WHO classification for neuroendocrine tumors arising in the GI tract.

Differentiation	Grade	Mitotic count	Ki-67 index	Traditional	ENETS, WHO
Well differentiated	Low grade (G1)	<2 per 10 HPF	<3%	Carcinoid, islet cell, pancreatic (neuro)endocrine tumor	Neuroendocrine tumor, G1
	Intermediate grade (G2)	2 to 20 per 10 HPF	3 to 20%	Carcinoid, atypical carcinoid, islet cell, pancreatic (neuro)endocrine tumor	Neuroendocrine tumor, G2

Poorly differentiated	High grade (G3)	>20 per 10 HPF	>20%	Small-cell carcinoma	Neuroendocrine carcinoma, G3, small cell
				Large-cell neuroendocrine carcinoma	Neuroendocrine carcinoma, G3, large cell

ENETS: European Neuroendocrine Tumor Society; WHO: World Health Organization; HPF: high-power fields.
